# Randomized Evaluation of Anagliptin vs Sitagliptin On low-density lipoproteiN cholesterol in diabetes (REASON) Trial: A 52-week, open-label, randomized clinical trial

**DOI:** 10.1038/s41598-019-44885-x

**Published:** 2019-06-12

**Authors:** Takeshi Morimoto, Ichiro Sakuma, Mio Sakuma, Akihiro Tokushige, Masahiro Natsuaki, Tomohiro Asahi, Michio Shimabukuro, Takashi Nomiyama, Osamu Arasaki, Koichi Node, Shinichiro Ueda

**Affiliations:** 1 Department of Clinical Epidemiology, Hyogo College of Medicine, 1-1 Mukogawa, Nishinomiya, Hyogo 663-8501 Japan; 2 Caress Sapporo Hokko Memorial Clinic N-27, E-8, 1-15, Higashi, Sapporo, Hokkaido 065-0027 Japan; 3 Department of Pharmacology and Therapeutics, University of the Ryukyus, 207 Uehara, Nishihara, Okinawa 903-0215 Japan; 4 Department of Cardiovascular Medicine, Saga University, 5-1-1 Nabeshima, Saga, 849-8501 Japan; 5 Department of Cardiology, Naha City Hospital, 2-31-1 Furujima, Naha, Okinawa 902-8511 Japan; 6 Department of Diabetes, Endocrinology and Metabolism, Fukushima Medical University, 1 Hikarigaoka, Fukushima, 960-1295 Japan; 7 Department of Endocrinology and Diabetes Mellitus, Fukuoka University, 7-45-1 Nanakuma, Jyonan, Fukuoka 814-0180 Japan; 8 Department of Cardiology, Tomishiro Central Hospital, 25 Ueda, Tomigusuku, Okinawa 901-0243 Japan

**Keywords:** Type 2 diabetes, Dyslipidaemias

## Abstract

Additional reductions in low-density lipoprotein-cholesterol (LDL-C) via antidiabetic therapies should be considered in statin-using patients with sub-optimal LDL-C levels. We compared the efficacy of anagliptin and sitagliptin, two antidiabetic therapies, in reducing LDL-C in type 2 diabetic patients. A randomized, open-label, parallel-group trial was conducted at 17 centres in Japan between April 2015 and January 2018. Adults (age ≥20 years) with type 2 diabetes, any atherosclerotic vascular lesions, and statin prescriptions were included. Anagliptin or sitagliptin were administered for 52 weeks. Primary and secondary endpoints were changes in LDL-C and haemoglobin A1C (HbA1c) levels, respectively. We assessed the superiority (primary endpoint) and non-inferiority (secondary endpoint) of anagliptin over sitagliptin. This study was registered at Clinicaltrials.gov (NCT02330406). Of 380 participants, 353 were eligible and randomized. Mean participant age was 68 years, and 61% were males. Baseline median LDL-C and HbA1c were 108 mg/dL and 6.9%, respectively. Changes in LDL-C were −3.7 mg/dL with anagliptin and +2.1 mg/dL with sitagliptin at 52 weeks, and the estimated treatment difference was a significant −4.5 mg/dL (P = 0.01 for superiority). Changes in HbA1c were +0.02% with anagliptin and +0.12% with sitagliptin (P < 0.0001 for non-inferiority). Overall, anagliptin was superior to sitagliptin in lowering LDL-C without deteriorating HbA1c.

## Introduction

The reduction of low-density lipoprotein-cholesterol (LDL-C) via statin prescription is a well-established regimen to prevent cardiovascular events in high-risk patients^1,2^. A recent large-scale clinical trial in Japan showed that high-dose statin administration significantly reduced cardiovascular events more so than low-dose statin prescription in patients with stable coronary disease. This high-dose statin therapy also reduced the risk of composite cardiovascular events by 19% at a median 3.9 year follow-up, with a 14.7 mg/dL decrease in LDL-C^3^. Diabetes mellitus is one of the largest risk factors for cardiovascular events, with a higher incidence of cardiovascular events when dyslipidaemia and diabetes coexist^4–6^. Because of this risk, physicians should manage dyslipidaemia more strictly in diabetic than non-diabetic patients. The American Diabetes Association recommends statin therapy for diabetic patients over 40 years of age regardless of their LDL-C levels or presence of other cardiovascular comorbidities^6^.

The American Heart Association and American Diabetes Association do not recommend a target LDL-C level to prevent cardiovascular events in patients with diabetes^6^, but the European Society of Cardiology recommends that LDL-C be less than 100 mg/dL^7^. Diabetic patients are resistant to statin therapy when compared to non-diabetic patients^8^, and diabetes-induced inflammation was reported to increase intestinal cholesterol absorption^9^. In addition, a recent meta-analysis indicated that statin use was associated with an increased risk of type 2 diabetes development^10^. If additional LDL-C reduction can be obtained via antidiabetic drug and statin co-therapy in diabetic patients, such a regimen should be considered for patients exhibiting suboptimal LDL-C reduction with statins. Anagliptin, a type of dipeptidylpeptidase-4 (DPP-4) inhibitor, was shown to reduce LDL-C by 9.5 mg/dL in a phase III trial regardless of statin administration^11^. An experimental study suggested that anagliptin reduces LDL-C by inhibiting cholesterol absorption in the small intestine and cholesterol synthesis in the liver^12^. However, it is unknown if these responses are a class effect among DPP-4 inhibitors. Here, we conducted a randomized, controlled trial to evaluate the effect of anagliptin and sitagliptin, another DPP-4 inhibitor, on changes in LDL-C levels in patients with type 2 diabetes, dyslipidaemia, and existing atherosclerotic vascular lesions currently prescribed statins.

## Results

### Participants

**Figure 1:**
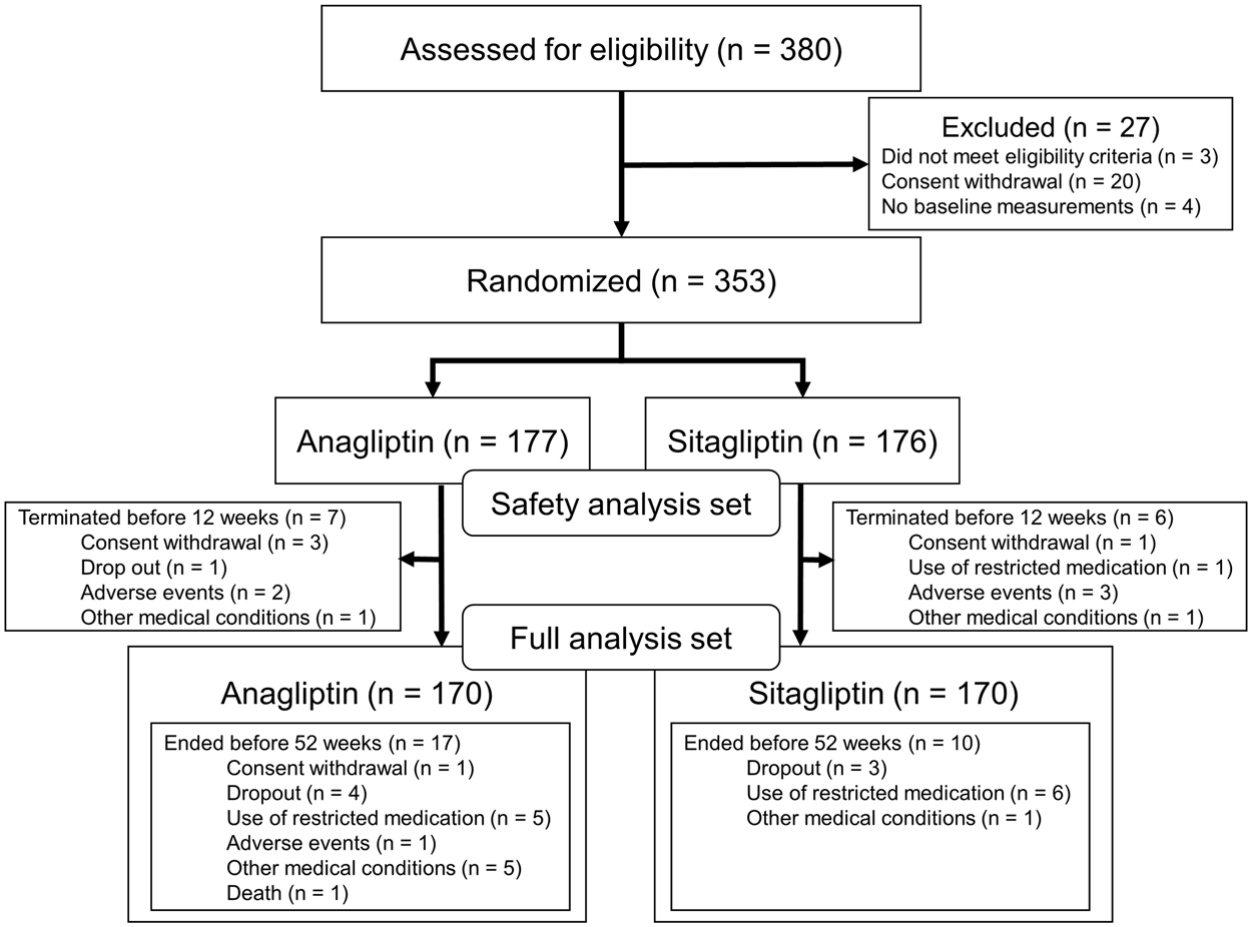
Participants Flow Chart.

Of the 380 participants screened for eligibility, 27 participants were excluded (Fig. 1). We randomized the remaining 353 participants to receive either anagliptin (n = 177) or sitagliptin (n = 176). A total of 153 (86.4%) and 160 (90.9%) participants receiving anagliptin and sitagliptin, respectively, completed 52 weeks of treatment. Ninety-three percent (142/153) of anagliptin users and 96% (154/160) of sitagliptin users followed their treatment regimen exactly as directed. Baseline patient characteristics were comparable between treatment groups (Table 1). Eighty-two percent of participants took DPP-4 inhibitors prior to study enrolment. Median baseline LDL-C was 112 mg/dL and 106 mg/dL in the anagliptin and sitagliptin groups, respectively.

**Table 1:** Baseline Characteristics.

Characteristics	All patients	Anagliptin	Sitagliptin
	(n = 353)	(n = 177)	(n = 176)
Age-years, mean (SD)	68 (10)	68 (10)	68 (9)
Gender male, n (%)	214 (61)	110 (62)	104 (59)
Body mass index-kg/m^2^, mean (SD)	26.2 (3.8)	26.5 (4.0)	25.9 (3.5)
Waist circumference-cm, mean (SD)	93.2 (10.5)	93.7 (11.2)	92.8 (9.6)
Systolic blood pressure-mmHg, mean (SD)	133 (16)	134 (16)	132 (16)
Diastolic blood pressure-mmHg, mean (SD)	72 (12)	74 (12)	71 (11)
Heart rate-bpm, mean (SD)	73 (11)	74 (12)	71 (11)
History of acute coronary syndrome, n (%)	88 (25)	53 (30)	35 (20)
History of acute myocardial infarction, n (%)	61 (17)	37 (21)	24 (14)
Unstable angina, n (%)	37 (10)	22 (12)	15 (9)
History of stable angina, n (%)	75 (21)	36 (20)	39 (22)
History of primary coronary intervention, n (%)	127 (36)	66 (37)	61 (35)
History of coronary artery bypass graft, n (%)	26 (7)	14 (8)	12 (7)
History of coronary lesion from previous coronary angiogram, n (%)	112 (32)	55 (31)	57 (32)
Coronary stenosis, n (%)	112 (32)	55 (31)	57 (32)
Coronary plaque, n (%)	46 (13)	25 (14)	21 (12)
History of coronary lesion from previous coronary CT, n (%)	72 (20)	37 (21)	35 (20)
Coronary stenosis, n (%)	55 (16)	27 (15)	28 (16)
Coronary plaque, n (%)	37 (10)	25 (14)	12 (7)
Coronary calcification, n (%)	59 (17)	29 (16)	30 (17)
History of peripheral arterial disease, n (%)	46 (13)	24 (14)	22 (13)
Aortic dissection, n (%)	1 (0.3)	1 (0.6)	0 (0)
Aortic aneurysm, n (%)	4 (1.1)	3 (1.7)	1 (0.6)
Arteriosclerosis obliterans, n (%)	21 (6)	12 (7)	9 (5)
Carotid artery stenosis, n (%)	27 (8)	12 (7)	15 (9)
History of stroke, n (%)	57 (16)	29 (16)	28 (16)
Ischemic stroke, n (%)	52 (15)	26 (15)	26 (15)
Intracranial hemorrhage, n (%)	3 (0.9)	1 (0.6)	2 (1.1)
Hemorrhagic stroke, n (%)	0 (0)	0 (0)	0 (0)
Subarachnoid hemorrhage, n (%)	2 (0.6)	2 (1.1)	0 (0)
History of transient ischemic attack, n (%)	7 (1.98)	4 (2)	3 (1.7)
History of abnormal ankle brachial index, n (%)	32 (9)	16 (9)	16 (9)
History of intima media thickness, n (%)	244 (69)	117 (66)	127 (72)
History of hypertension, n (%)	270 (76)	137 (77)	133 (76)
Family history of coronary artery disease, n (%)	55 (16)	21 (12)	34 (19)
Smoker, n (%)	195 (55)	92 (52)	103 (59)
Current smoker, n (%)	54 (15)	30 (17)	24 (14)
Past smoker, n (%)	141 (40)	62 (35)	79 (45)
Drinker, n (%)	149 (42)	75 (42)	74 (42)
Current drinker, n (%)	52 (15)	27 (15)	25 (14)
Occasional drinker, n (%)	97 (27)	48 (27)	49 (28)
History of drug allergy, n (%)	22 (6)	10 (6)	12 (7)
Dipeptidyl peptidase-4 inhibitors before enrollment, n (%)	290 (82)	145 (82)	145 (82)
Alogliptin, n (%)	48 (14)	21 (12)	27 (15)
Anagliptin, n (%)	7 (2)	2 (1)	5 (3)
Linagliptin, n (%)	34 (10)	19 (11)	15 (9)
Saxagliptin, n (%)	9 (3)	6 (3)	3 (2)
Sitagliptin, n (%)	111 (31)	52 (29)	59 (34)
Teneligliptin, n (%)	16 (5)	8 (5)	8 (5)
Vildagliptin, n (%)	65 (18)	37 (21)	28 (16)
Anti-diabetic drugs, n (%)	243 (69)	124 (70)	119 (68)
Biguanides, n (%)	171 (48)	87 (49)	84 (48)
Thiazolidines, n (%)	56 (16)	25 (14)	31 (18)
Sulfonylureas, n (%)	83 (24)	46 (26)	37 (21)
Alpha-glucosidase inhibitors, n (%)	56 (16)	28 (16)	28 (16)
Glinides, n (%)	9 (3)	7 (4)	2 (1.1)
Insulins, n (%)	28 (8)	13 (7)	15 (9)
Sodium-dependent glucose transporter 2 inhibitors, n (%)	45 (13)	27 (15)	18 (10)
Anti-dyslipidemia drugs, n (%)	353 (100)	177 (100)	176 (100)
Statins, n (%)	353 (100)	177 (100)	176 (100)
Anion exchangers, n (%)	0 (0)	0 (0)	0 (0)
Ezetimibes, n (%)	30 (9)	18 (10)	12 (7)
Probucols, n (%)	2 (0.6)	2 (1.1)	0 (0)
Fibrates, n (%)	20 (6)	11 (6)	9 (5)
Eicosapentaenoic acids, n (%)	31 (9)	16 (9)	15 (9)
Nicotinic acids, n (%)	1 (0.3)	0 (0)	1 (0.6)
Sterols, n (%)	0 (0)	0 (0)	0 (0)
Aspirin, n (%)	153 (43)	84 (47)	69 (39)
Ticlopidine, n (%)	13 (4)	7 (4)	6 (3)
β-blockers, n (%)	84 (24)	44 (25)	40 (23)
Angiotensin-converting enzyme inhibitors, n (%)	33 (9)	22 (12)	11 (6)
Angiotensin II receptor blockers, n (%)	177 (50)	87 (49)	90 (51)
Calcium channel blockers, n (%)	164 (46)	85 (48)	79 (45)
Nitroglycerines, n (%)	19 (5)	12 (7)	7 (4)
Diuretics, n (%)	52 (15)	30 (17)	22 (13)
Aldosterone antagonists, n (%)	14 (4)	8 (5)	6 (3)
White blood cell-mm^3^, median [IQR]	6100 [5200–7200]	6365 [5325–7500]	5900 [4900–7000]
Red blood cell- × 10^4^mm^3^, median [IQR]	460 [426–490]	465 [426–496]	453 [426–484]
Hemoglobin-g/dL, median [IQR]	13.9 [13.0–15.1]	14.0 [13.2–15.2]	13.9 [12.7–14.8]
Hematocrit-%, median [IQR]	41.9 [38.9–44.8]	42.6 [39.1–45.4]	41.6 [38.5–44.0]
Platelet- × 10^4^mm^3^, median [IQR]	20.8 [17.9–24.8]	21.1 [17.4–24.7]	20.8 [18.3–24.9]
Fasting blood glucose-mg/dL, median [IQR]	133 [116–158]	136 [115–161]	128 [117–153]
Aspartate transaminase-IU/L, median [IQR]	22 [18–28]	24 [18–30]	20 [18–26]
Alanine aminotransferase-IU/L, median [IQR]	21 [15–30]	22 [16–34]	19 [15–26]
γ- glutamyl transpeptidase-IU/L, median [IQR]	26 [18–41]	30 [18–46]	25 [18–36]
Creatine kinase-IU/L, median [IQR]	99 [71–138]	93 [69–136]	102 [73–144]
Blood urea nitrogen-mg/dL, median [IQR]	15.9 [12.9–20.0]	15.3 [12.8–19.7]	16.1 [13.0–20.0]
Creatinine-mg/dL, median [IQR]	0.80 [0.66–0.97]	0.78 [0.66–0.97]	0.81 [0.66–0.98]
Estimated glomerular filtration rate-mL/min/1.73 m^2^, median [IQR]	67.69 [55.06–80.59]	68.64 [55.31–83.17]	66.80 [54.32–78.77]
Hemoglobin A1c-%, median [IQR]	6.9 [6.4–7.4]	7.0 [6.4–7.6]	6.8 [6.4–7.3]
LDL cholesterol-mg/dL, median [IQR]	108 [96–122]	112 [97–123]	106 [94–121]
Total cholesterol-mg/dL, median [IQR]	185 [169–204]	188 [171–207]	183 [167–199]
HDL cholesterol-mg/dL, median [IQR]	52 [45–61]	52 [45–60]	53 [45–62]
Triglycerides-mg/dL, median [IQR]	120 [86–167]	129 [95–186]	114 [80–159]

### Primary endpoint

**Figure 2:**
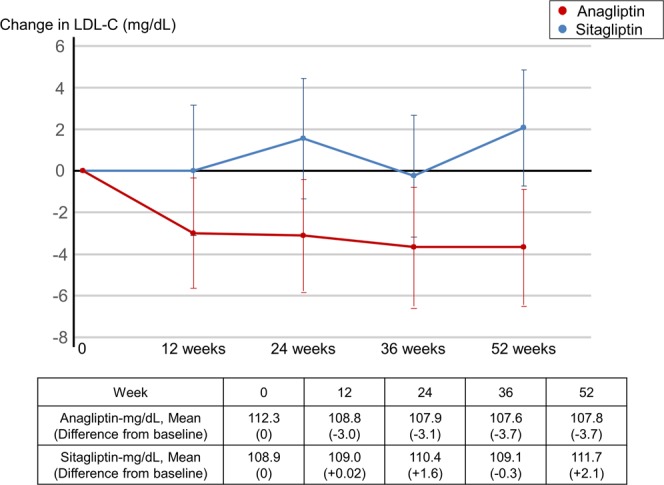
Change in Low-density Lipoprotein-cholesterol. LDL-C: low-density lipoprotein-cholesterol.

Mean LDL-C decreased by 3.7 mg/dL with anagliptin (Fig. 2). This effect did not occur with 52-weeks of sitagliptin treatment (estimated treatment difference between anagliptin and sitagliptin was −4.5 mg/dL (95% CI −8.0, −1.0; p = 0.01 for superiority). The reduction in LDL-C with anagliptin treatment was achieved at 12 weeks and continued until the end of the study.

### Subgroup analyses of primary endpoint

**Figure 3:**
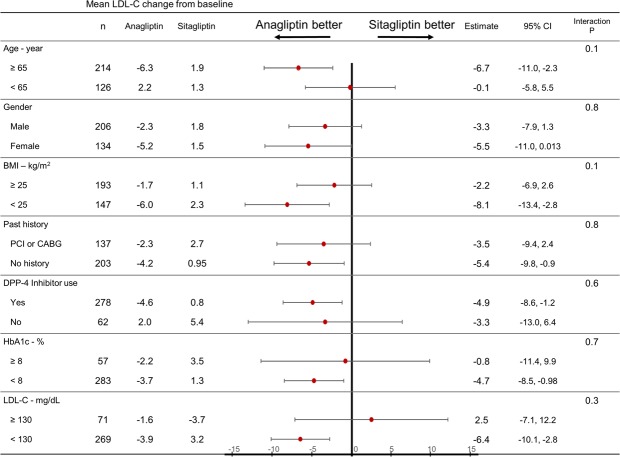
Primary endpoint subgroup analyses. LDL-C: Low density lipoprotein-cholesterol. BMI: body mass index. PCI: Percutaneous coronary intervention. CABG: Coronary artery bypass surgery. DPP-4: dipeptidylpeptidase-4. HbA1c: hemoglobin A1c.

The effects of anagliptin and sitagliptin on LDL-C reduction were generally similar in the pre-specified subgroups, and the interaction p-values were not significantly different (Fig. 3). Patients displaying a greater beneficial effect included the elderly (≥65 years), previous DPP-4 inhibitor users, patients without a history of coronary revascularization, and patients with lower baseline HbA1c (<8.0%), LDL-C (<130 mg/dL), and body mass index (BMI) (<25 kg/m^2^).

### Important secondary endpoint

**Figure 4:**
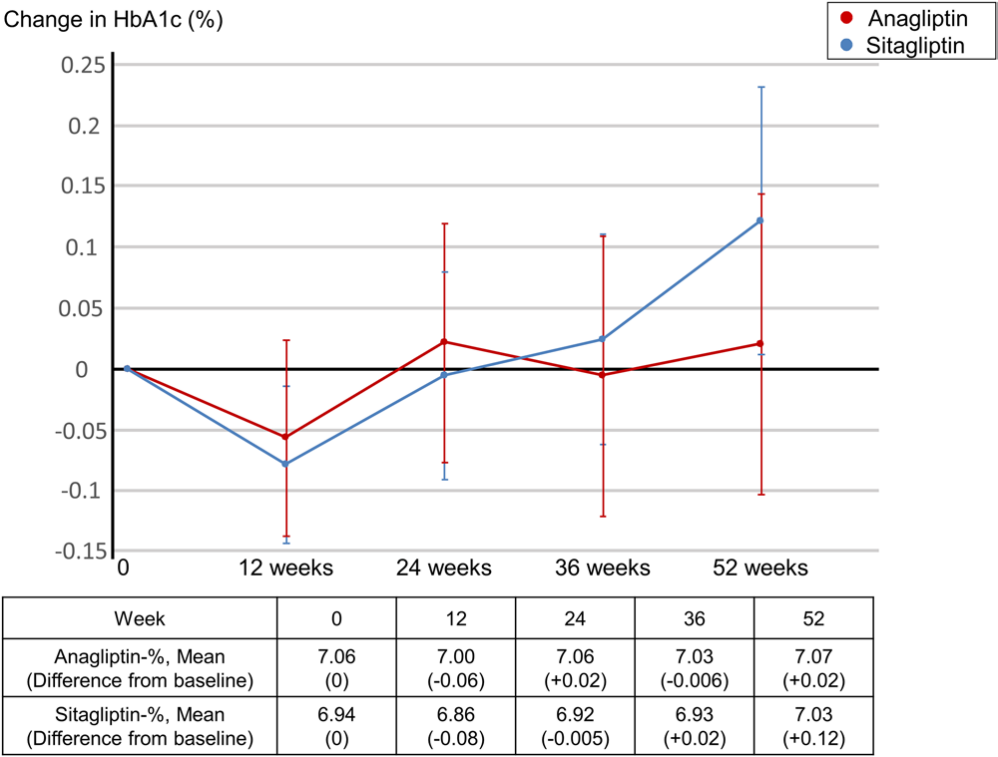
Changes in Hemoglobin A1c. HbA1c: hemoglobin A1c.

Mean haemoglobin A1c (HbA1c) was observed to fluctuate slightly; however, the level did not significantly change over time (Fig. 4). The difference in HbA1c from baseline to study completion was +0.02% and +0.12% with anagliptin and sitagliptin, respectively. The estimated treatment difference between anagliptin and sitagliptin was −0.036% (95% CI −0.191, 0.119; p < 0.0001 for non-inferiority, p = 0.65 for superiority).

### Secondary endpoints

At 52 weeks, mean total cholesterol (TC) decreased by 3.7 mg/dL (190.5 to 186.2) with anagliptin but increased by 4.8 mg/dL (185.7 to 191.3) with sitagliptin. This difference was statistically significant (p = 0.0007). Mean high density lipoprotein cholesterol (HDL-C) decreased from 53.9 to 53.3 mg/dL (−0.4) with anagliptin treatment but increased from 54.7 to 56.0 mg/dL (+1.4) with sitagliptin treatment. This difference was also statistically significant (p = 0.02). Mean triglycerides (TG) slightly increased with both anagliptin from 147.8 to 155.2 mg/dL (+3.4) and sitagliptin from 128.9 to 135.8 mg/dL (+7.2) treatment, but this difference was not statistically different at 52 weeks (p = 0.62).

### Safety outcomes

Both anagliptin and sitagliptin were well tolerated and only 3 terminations of use occurred in each group due to adverse events (AEs). All safety outcomes are summarized in Table 2. Cardiovascular events occurred in 6 and 5 participants in the anagliptin and sitagliptin groups, respectively. Pancreatic cancer occurred in one anagliptin user, and pancreatitis was not reported. No hypoglycaemic events were reported in either group.

**Table 2:** Safety outcomes.

Safety outcomes	Anagliptin	Sitagliptin
	(n = 177)	(n = 176)
Cardiovascular events, n	6	5
Acute coronary syndrome, n	1	1
Congestive heart failure, n	2	3
Cerebral stroke, n	1	0
Subdural hematoma, n	2	0
Peripheral arterial disease, n	0	1
Malignancy, n	5	1
Pancreatic cancer, n	1	0
Colorectal cancer, n	3	1
Lung cancer, n	1	0
Benign gastrointestinal symptoms, n	4	4
Liver dysfunction, n	1	0
Infectious diseases, n	6	2
Other adverse events^*^, n	3	5

^*^Other adverse events included diabetic foot, knee osteoarthrosis, vertebral compression fracture, arthralgia, hyperkalemia, palpitation, edema, and skin eruption.

## Discussion

To the best of our knowledge, this is the first study specifically designed to investigate the effect of DPP-4 inhibitors on lowering LDL-C in patients with diabetes. The 52-week twice-daily oral administration of 100 mg anagliptin lowered LDL-C by 3.7 mg/dL in patients with well-controlled diabetes and receiving statins without deteriorating the control of diabetes and other lipids. This effect was significantly greater (4.5 mg/dL) than the oral administration of 50 mg sitagliptin once daily, and was consistent in several of the clinically meaningful subgroups.

The results of this study replicate previously reported findings for the magnitude of LDL-C reduction. In one study, the reduction in LDL-C by anagliptin was 4 mg/dL in patients with a baseline LDL-C less than 140 mg/dL^11^. In our study, the median [IQR] of baseline LDL-C was 108 [96–122]. Patients with a relatively low baseline LDL-C experienced the same magnitude of effect, with a 3.7 mg/dL reduction observed in the current investigation. Another recent one-arm pre-post observation showed a 7.3 mg/dL reduction in LDL-C with anagliptin treatment at 24 weeks, with a larger magnitude of LDL-C reduction in patients with higher baseline LDL-C^13^. This reduced LDL-C was not consistent when a DPP-4 inhibitor (linagliptin, sitagliptin, or vildagliptin) was used^14–16^. Based on this information, we utilized sitagliptin, the most frequently prescribed DPP-4 inhibitor, as an active control instead of a placebo. We were interested to evaluate whether the effect of anagliptin on LDL-C reduction could be duplicated by another DPP-4 inhibitor.

The mechanism of LDL-C reduction via anagliptin has been previously reported. Anagliptin inhibits cholesterol absorption in the small intestine and cholesterol synthesis in the liver^11,12,17^. Our study shed light on the particular effect of anagliptin on LDL-C,and this effect seemed different than the effects of other DPP-4 inhibitors. As such, further investigations on the effect of each DPP-4 inhibitor on the reduction of LDL-C and other lipids should be mandatory.

The 4.5 mg/dL difference in LDL-C reduction may seem too small to prevent cardiovascular events. However, patients enrolled in this study had already received statin therapy; thus, excess reductions should theoretically be small. However, this study clearly demonstrated a small but additional reduction in LDL-C using anagliptin in statin-using patients with type 2 diabetes and risk factor of cardiovascular events. While these results seem promising, further large-scale clinical trials should be conducted to evaluate the association between the small LDL-C reduction and the prevention of cardiovascular events in anagliptin users.

This study has a few limitations. First, LDL-C reduction was a surrogate measure of cardiovascular events. As such, the impact of the 4.5 mg/dL difference was unclear. A previous phase III trial showed a 25 mg/dL reduction in LDL-C with anagliptin therapy in patients with a baseline LDL-C ≥ 140 mg/dL^11^. This study provides additional evidence for the benefit of concomitant drug therapy in the presence of diabetes and other existing risk factors for cardiovascular events. Second, because our goal was to verify the effects of anagliptin and sitagliptin in a real-world setting, we performed an open-label study instead of utilizing a placebo. The placebo effect and ascertainment bias were less likely to occur, as the primary and secondary endpoints were serum measurements, which were obtained as scheduled and measured at the core laboratory. Finally, the safety outcomes were not well attested as the sample size was not large enough to confirm the low-frequency events. Therefore, our findings should be validated using a large-scale study.

To conclude, anagliptin reduces LDL-C without affecting HbA1c control in statin-using patients with type 2 diabetes and risk factors for cardiovascular events. The magnitude of LDL-C reduction was statistically significant, but its clinical impact is uncertain. Therefore, further studies to explore the impact of this magnitude on the prevention of cardiovascular events in patients with type 2 diabetes and risk factor of cardiovascular events are mandatory.

## Methods

### Trial design and participants

This Randomized Evaluation of Anagliptin versus Sitagliptin On low-density lipoproteiN cholesterol in diabetes (REASON) Trial was a multicentre, randomized, open-label, active-controlled, parallel-group trial. Seventeen centres participated in this trial. Patient enrolment began in April 2015 and ended in January 2017. The rationale and study design are published elsewhere^18^.

Eligible participants were adults (≥20 years of age) diagnosed with type 2 diabetes (HbA1c 6.0–10.5% (7.0–10.5% for those not currently prescribed the investigational drug)). Participants were also treated with diet and exercise alone or in combination with hypoglycaemic agents, had existing atherosclerotic vascular lesions (stenotic lesions or plaques of ≥25% of the arterial diameter in past coronary angiography or CT; coronary artery calcification in past coronary CT; past history of acute coronary syndrome; past history of percutaneous coronary intervention (PCI) or coronary artery bypass surgery (CABG); past history of ischemic cerebral infarction, cerebral haemorrhage or transient ischemic attack; past history of peripheral artery or aortic disease; past ankle-brachial index ≤0.9; presence of carotid artery plaque or max intima-media thickness ≥1.1 mm in past carotid duplex), treated with statins for dyslipidaemia for ≥8 weeks, and a documented LDL-C ≥ 100 mg/dL in ≥1 measurement after statin use. Key exclusion criteria were type 1 diabetes, TG ≥ 400 mg/dL in a past fasting blood sample, pregnant/potentially pregnant/ lactating women, severe infection, surgery, serious trauma, serum creatinine ≥2.4 mg/dL for men or ≥2.0 mg/dL for women, and use of glucagon-like peptide-1 receptor agonists.

This study was conducted in accordance with the Declaration of Helsinki and the Ethical Guidelines for Medical and Health Research Involving Human Subjects in Japan. The institutional review boards at the University of the Ryukyus (No. 731) and each participating centre approved this study, and all patients or their legally authorized representatives provide written, informed consent prior to randomization. Other trial oversights are described elsewhere^18^. This trial was registered at Clinicaltrials.gov on 05/01/2015 (NCT02330406).

### Randomization and intervention

We used an electronic data capture (EDC) system to register and randomize participants and collect data. Participants randomly received anagliptin or sitagliptin in a 1:1 ratio. Randomization was performed centrally through the EDC system, with a stochastic minimization algorithm to balance treatment assignment within the strata of centres: HbA1c (≥8.0%, <8.0%), use of DPP-4 inhibitors prior to trial registration, sex, BMI (≥25 kg/m^2^, <25 kg/m^2^), and LDL-C (≥130 mg/dL, <130 mg/dL).

Participants in the anagliptin group were prescribed 100 mg anagliptin (orally; twice daily) for 52 weeks. If effects were insufficient, the dose could be increased to 200 mg orally twice daily. Participants in the sitagliptin group were prescribed 50 mg (orally; once daily) for 52 weeks. If effects were insufficient, the dose could be increased to 100 mg per day. These anagliptin and sitagliptin doses are considered equivalent in Japan. If participants used any antidiabetic drugs other than DPP-4 inhibitors at the start of the trial, the study drug was administered concomitantly, and the original antidiabetic drugs were not replaced. Treatment assignment was not concealed from participants or physicians.

During the trial period, hypoglycaemic agents and anti-dyslipidaemia drugs (statins, ezetimibe, anion exchange resins, fibrates, and eicosapentaenoic acid) were not added, and their dosages were not changed. A change in insulin dose was not considered a change in hypoglycaemic agents. The need for other therapy was determined by the physician in charge; however, changes in other medications with possible effects on the study outcome were prohibited. Clinical research coordinators regularly monitored participants and their physicians to ensure adherence to the study medication/dose at every visit. If cross-over was found, the participants were removed as per protocol.

### Endpoints

The primary endpoint was the change in LDL-C, which was calculated based on the Friedewald (F) equation^19^. An important secondary endpoint was change in HbA1c. We measured LDL-C and HbA1c at baseline, and at 12, 24, 36, and 52 weeks. These measurements were analyzed at the core laboratory (SRL Inc., Tokyo, Japan). TC, HDL-C and TG were measured at the same study timepoints and analyzed at the same core laboratory. Safety outcomes were all-cause mortality, cardiovascular events, and other AEs over the 52-week period. Cardiovascular events included coronary heart disease, congestive heart failure, ischemic and haemorrhagic stroke, and peripheral arterial disease. Other AEs were described as any symptoms reported by the clinical research coordinators or the physician in charge. Participant information was obtained from clinical research coordinators or physicians.

### Measurements

Clinical characteristics included age, sex, height, waist circumference, body weight, blood pressure, heart rate, past medical history, smoking status, alcohol consumption, drug allergies, and use of concomitant drugs. Blood pressure, heart rate, and compliance with study drug were monitored at 12, 24, 36, and 52 weeks. Height, waist circumference, and body weight were also measured at 52 weeks.

Blood glucose, red and white blood cell counts, haemoglobin, haematocrit, platelet count, aspartate transaminase, alanine aminotransferase, γ-glutamyl transpeptidase, creatine kinase, blood urea nitrogen, and creatinine were measured locally at baseline, 12, 24, 36, and 52 weeks to assess AEs.

### Statistical analysis

The primary hypothesis was that anagliptin was significantly superior to sitagliptin in the change in LDL-C after 52 weeks of treatment. Thus, the null hypothesis was that both anagliptin and sitagliptin result in equal changes in LDL-C levels, while the alternative hypothesis was that the sitagliptin and anagliptin result in different changes in LDL-C. We also simultaneously hypothesized that anagliptin was non-inferior to sitagliptin for the important secondary endpoint of HbA1c change, with the non-inferiority margin of HbA1c as 0.3%. For baseline data, missing data were not input, and data was only analyzed using the existing data. Owing to the short enrolment and follow-up periods and the estimated low risk of AEs, no interim analyses were planned. The details of statistical analysis are described in a previous report, and the statistical analysis plan (SAP) was fixed before data analysis^18^.

To calculate sample size, it was assumed that reductions in LDL-C after 52 weeks would be similar to a previous report where anagliptin resulted in an LDL-C decrease of 9.5 mg/dL, whereas sitagliptin resulted in a decrease of 0.97 mg/dL, with a standard deviation (SD) of 21.6 mg/dL for both treatments^18^. With a two-sided alpha of 0.05 and power of 0.8, the sample size was calculated as 102 for each group. Considering a dropout rate of 30%, the total sample size was set to 300. This total sample size provided a power of 0.8 for HbA1c on the assumption that the SD for HbA1c in the anagliptin group, based on a previous clinical trial, was 0.91. The non-inferiority margin was 0.3 with a one-sided alpha of 0.025.

Efficacy analyses were performed for the full analysis set, which included participants who received an allocated treatment and provided assessable outcome data. Safety data were evaluated on the safety analysis set, which included participants who received allocated treatment at least once. All analyses were conducted under the intention-to-treat principle.

Categorical variables were expressed as frequencies with percentages, and continuous variables were expressed as means with SDs or medians with inter-quartile ranges (IQRs). A mixed effects model for repeated measures was developed to estimate the estimated treatment difference of anagliptin against sitagliptin for changes in LDL-C and HbA1c; an unstructured covariance matrix was assumed for measurements within the same patient. The non-inferiority of the anagliptin group to the sitagliptin group in terms of HbA1c levels was examined with a non-inferiority margin of 0.3%. In these models, treatment allocation and time were treated as fixed effects, and 5 variables used in balancing factors at randomization were included as covariates. The same models were constructed to estimate the differences in the changes in LDL-C between the anagliptin and sitagliptin groups within the pre-specified subgroups, and the interaction p-values between treatment allocation and subgroup factors. The pre-specified subgroups included age (≥65 years, <65 years), gender, BMI (≥25 kg/m^2^, <25 kg/m^2^), the presence of a treatment for existing ischemic heart disease before enrolment (PCI or CABG), use of DPP-4 inhibitors prior to trial registration, HbA1c (≥8.0%, <8.0%), and LDL-C (≥130 mg/dL, <130 mg/dL).

We compared the differences from baseline to 52 weeks between groups by t-test for TC, HDL-C and TG. The number of safety outcomes is presented by group. As this study was not powered to compare safety outcomes between groups, tests were not performed to determine significance.

All statistical analyses were performed at the data centre (Institute for Clinical Effectiveness) by the study statisticians (Sakuma M and Morimoto T). JMP 13.1 (SAS Institute Inc, Cary, NC) and SAS 9.4 (SAS Institute Inc, Cary, NC) were used based on the SAP. All P values were two-sided, and P < 0.05 was considered significant. The one exception to these rules was the non-inferiority test for HbA1c, where a one-sided P < 0.025 was considered significant.

## Data Availability

The datasets generated during and/or analyzed during the current study are available from the corresponding author on reasonable request.
