# Author Correction: Randomized Evaluation of Anagliptin vs Sitagliptin On low-density lipoproteiN cholesterol in diabetes (REASON) Trial: A 52-week, open-label, randomized clinical trial

**DOI:** 10.1038/s41598-020-60644-9

**Published:** 2020-02-21

**Authors:** Takeshi Morimoto, Ichiro Sakuma, Mio Sakuma, Akihiro Tokushige, Masahiro Natsuaki, Tomohiro Asahi, Michio Shimabukuro, Takashi Nomiyama, Osamu Arasaki, Koichi Node, Shinichiro Ueda

**Affiliations:** 1 Department of Clinical Epidemiology, Hyogo College of Medicine, 1-1 Mukogawa, Nishinomiya, Hyogo 663-8501 Japan; 2 Caress Sapporo Hokko Memorial Clinic N-27, E-8, 1-15, Higashi, Sapporo, Hokkaido 065-0027 Japan; 3 Department of Pharmacology and Therapeutics, University of the Ryukyus, 207 Uehara, Nishihara, Okinawa 903-0215 Japan; 4 Department of Cardiovascular Medicine, Saga University, 5-1-1 Nabeshima, Saga, 849-8501 Japan; 5 Department of Cardiology, Naha City Hospital, 2-31-1 Furujima, Naha, Okinawa 902-8511 Japan; 6 Department of Diabetes, Endocrinology and Metabolism, Fukushima Medical University, 1 Hikarigaoka, Fukushima, 960-1295 Japan; 7 Department of Endocrinology and Diabetes Mellitus, Fukuoka University, 7-45-1 Nanakuma, Jyonan, Fukuoka 814-0180 Japan; 8 Department of Cardiology, Tomishiro Central Hospital, 25 Ueda, Tomigusuku, Okinawa 901-0243 Japan

Correction to: *Scientific Reports* 10.1038/s41598-019-44885-x, published online 12 June 2019

This Article contains errors. The calculation method of LDL-C, presented in Figure 2 and 3 is described as being based on the Friedewald (F) equation. However, the data presented was calculated based on the direct method.

The correct Figures 2 and 3 appear below as Figures 1 and 2.

**Figure 1:**
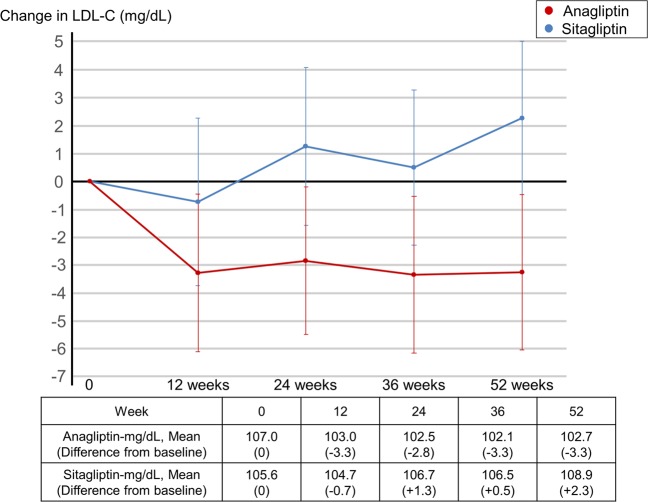
Change in Low-density Lipoprotein-cholesterol calculated by F equation. LDL-C: low-density lipoprotein-cholesterol.

**Figure 2:**
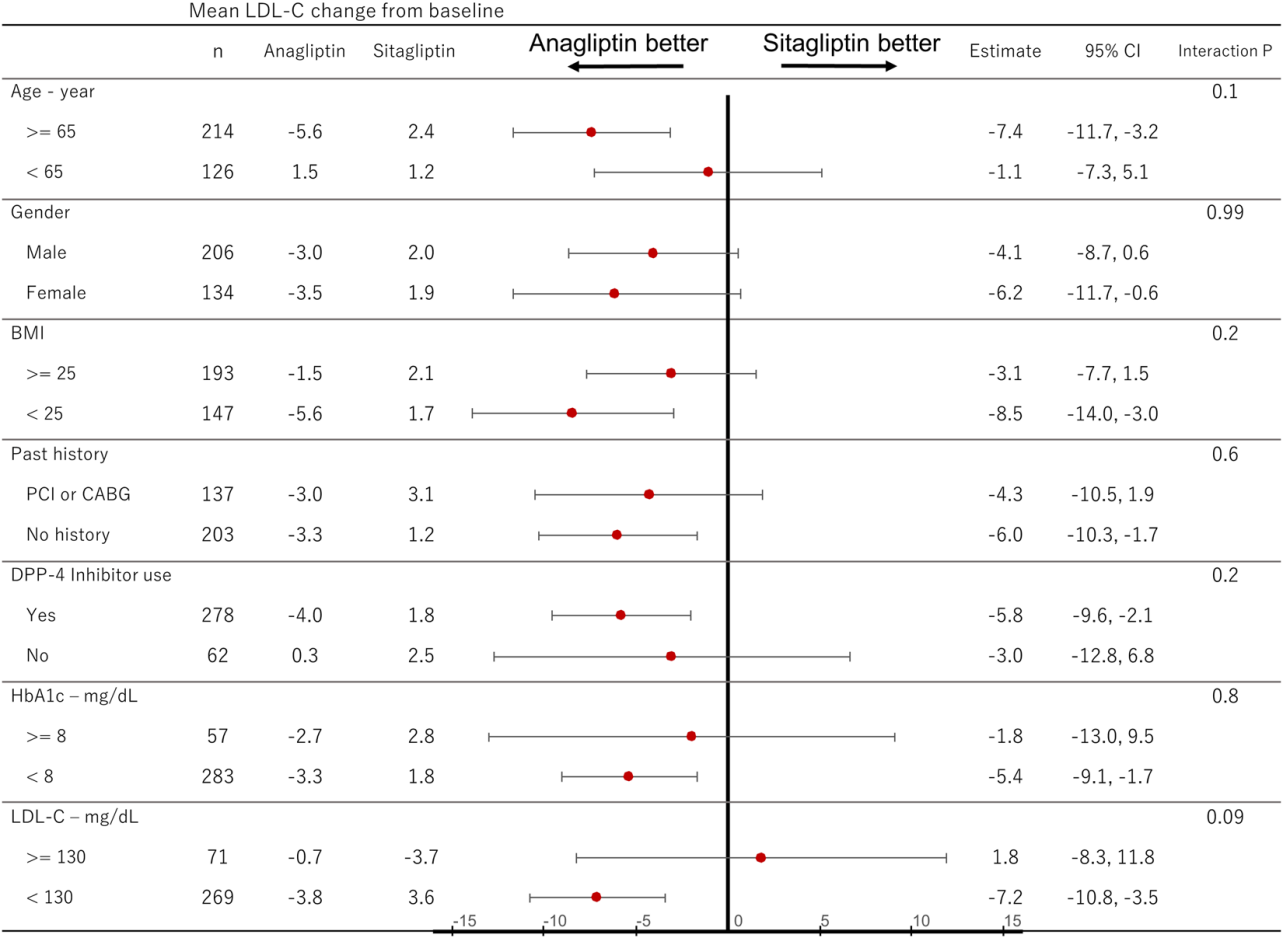
Primary endpoint subgroup analyses calculated by F equation. LDL-C: Low density lipoprotein-cholesterol. BMI: body mass index. PCI: Percutaneous coronary intervention. CABG: Coronary artery bypass surgery. DPP-4: dipeptidylpeptidase-4. HbA1c: hemoglobin A1c.

